# Revealing the causal role of immune cells in malignant neoplasms of the head and neck: insights from Mendelian randomization

**DOI:** 10.3389/fonc.2024.1435313

**Published:** 2024-11-29

**Authors:** En Zhou, MingHao Yuan, JiaYu Zhong, XuPing Xiao

**Affiliations:** ^1^ Department of Otolaryngology Head and Neck Surgery, Hunan Provincial People’s Hospital, The First Affiliated Hospital of Hunan Normal University, Changsha, China; ^2^ Department of Joint and Sports Medicine, The First Affiliated Hospital of Hunan Normal University, Hunan Provincial People’s Hospital, Changsha, China; ^3^ Department of Nuclear Medicine, Xiangya Hospital, Central South University, Changsha, Hunan, China

**Keywords:** head and neck, immunity, malignant neoplasm, mendelian randomisation, phenotype

## Abstract

**Background:**

Immune escape and immunosuppression play crucial roles in the onset and progression of head and neck malignant neoplasms (HNMN). However, previous studies on the relationship between immune cells and HNMN have yielded inconsistent results.

**Methods:**

In this study, we performed bidirectional two-sample Mendelian randomisation (MR) analyses using genome-wide association study (GWAS) and FinnGen databases to examine the association between 731 immune cell features and the risk of HNMN. We conducted sensitivity analyses to assess the robustness of the findings.

**Results:**

Subsequent to false discovery rate (FDR) correction, three immune cell phenotypes were found to have a significant correlation with the risk of HNMN: CD28−CD8+ absolute cells (AC) (inverse-variance weighted [IVW] using the multiplicative random effects model: OR [95%]: 1.325 [1.413 to 1.539], *P = 0.0002, Pfdr = 0.054*), CD3 on secreting Treg (IVW: OR [95%]: 0.887 [0.835 to 0.941], *P = 0.00007, Pfdr = 0.025*), and CD3 on resting Treg (IVW: OR [95%]: 0.891 [0.842 to 0.943], *P = 0.00006, Pfdr = 0.026*). The results of the sensitivity analysis were aligned with the primary findings. No statistically significant effects of HNMN on the immunophenotypes were observed.

**Conclusions:**

Our research indicates causal relationships among the three immune cell phenotypes and vulnerability to HNMN, providing new insights into immune infiltration within the HNMN tumour microenvironment and the development of immunotherapy drugs targeting checkpoint inhibitors of HNMN.

## 1 Introduction

Head and neck malignant neoplasms (HNMN) are common malignancies in humans, ranking sixth in incidence among malignant tumours worldwide (1). Head and neck squamous cell carcinoma (HNSCC) is the most common pathological subtype and can be classified into various types based on different anatomical sites, including pharyngeal cancer, oral cavity cancer, laryngeal cancer, AND sinonasal cancer (2). Due to the numerous similarities among the different subtypes of HNMN in terms of biological characteristics, pathogenic factors, immune microenvironment, and treatment methods, researchers often study them as a whole (2). Conventional treatments for HNMN include surgery, radiotherapy, and chemotherapy; however, the overall outcomes are not ideal. Between 70–80% of patients with HNSCC are initially diagnosed at a late stage (stage III or IV) (3, 4). After comprehensive treatment, the recurrence rate within 2 years for patients with advanced local disease is 40–60% (3). For recurrent or metastatic HNSCC, the median survival time following traditional chemotherapy or targeted therapy is only approximately 1 year (5, 6). Therefore, more effective diagnostic and treatment strategies are urgently needed for patients with HNSCC.

Recent studies have emphasised the critical role of immune evasion in the development of HNMN. Researchers have found the presence of regulatory T cell (Treg) infiltration, a reduced absolute count of T lymphocytes in the peripheral blood, and elevated levels of Tregs in patients with HNSCC (7, 8). Tregs are important inhibitory immune regulatory cells that play a key role in establishing an immunosuppressive microenvironment and promoting tumour progression (9). In the tumour microenvironment (TME) of HNSCC, the levels of immune regulatory factors such as programmed cell death protein 1 (PD-1), PD-L1, and T cell immunoglobulin and mucin domain 3 (TIM-3) are upregulated (10), alongside the interplay of various cytokines (11). These molecules play a critical role in establishing an immunosuppressive microenvironment, weakening the tumour-killing ability of cytotoxic T lymphocytes (CTLs), and promoting tumour cell proliferation in HNSCC. Moreover, CD4+ T lymphocytes, dendritic cells, macrophages, and NK cells all significantly contribute to the immune response within the tumour microenvironment of HNSCC (12). Thus, exploring the role and mechanisms of the immune system in HNMN may provide new insights for targeting immune checkpoint inhibition therapies, thereby improving treatment outcomes and prognosis for patients with HNMN. However, to date, the research findings on the relationship between immune cells and HNMN have been inconsistent, due to study design flaws, differences in experimental methods, small sample sizes, and other confounding factors.

Mendelian randomisation (MR) is primarily used in epidemiological research to infer causality. It uses genetic variants that have a significant impact on exposure as instrumental variables (IVs) to investigate a causal relationship between exposure and disease risk (13). The inheritance of genes is random, allowing an individual’s genome to serve as a natural experiment, similar to random assignment in experimental design. This randomness helps reduce the effects of confounding bias and reverse causation (14). The hypothesis of a correlation between immune cell characteristics and HNMN has been supported by several relationships identified in previous studies. In this study, a comprehensive two-sample MR analysis was performed to establish a causal relationship between immune cell signatures and HNMN. This study aims to identify new strategies for improving treatment outcomes for patients with HNMN, particularly through the use of immune checkpoint inhibitor therapies.

## 2 Materials and methods

### 2.1 Study design

**Figure 1:**
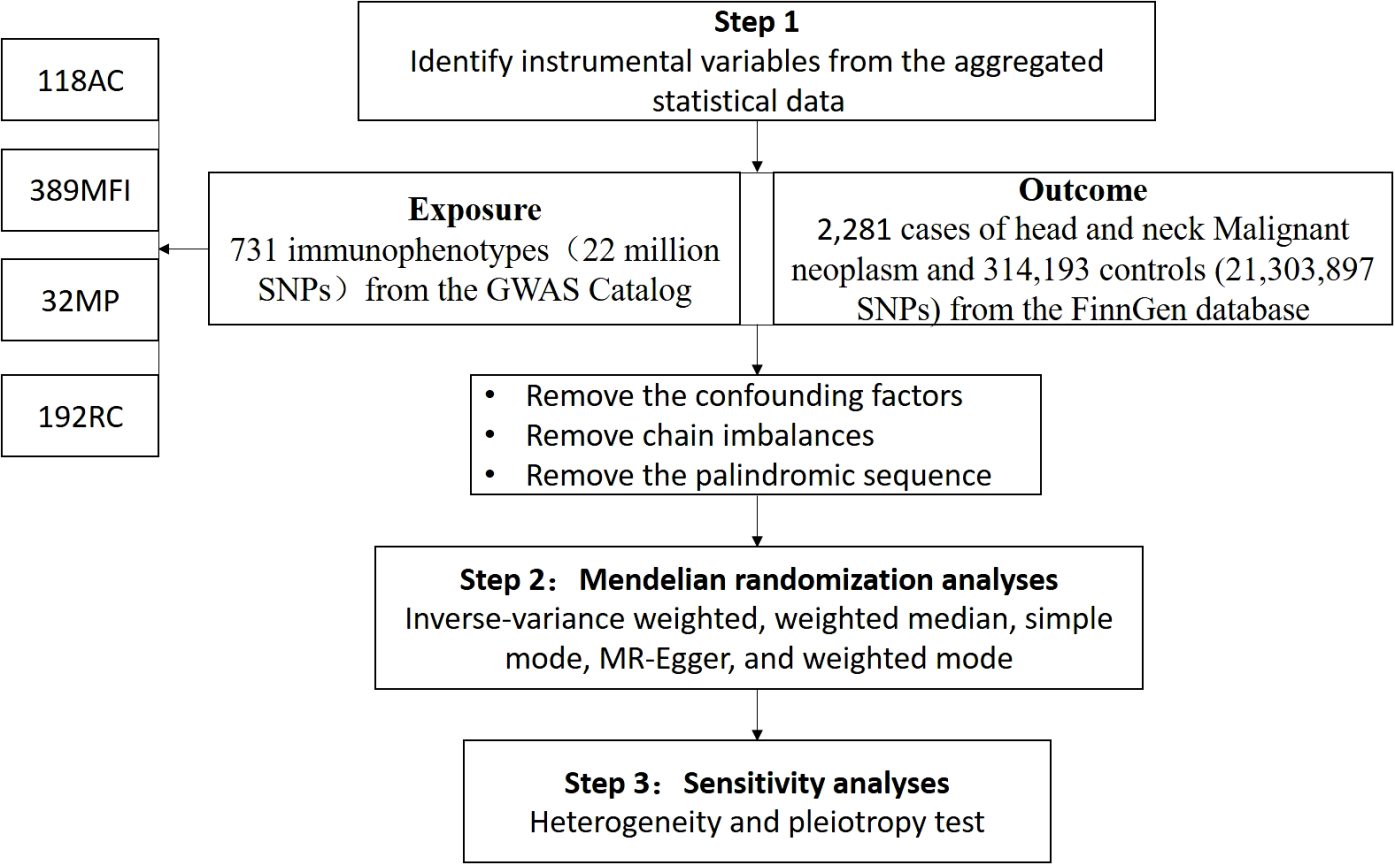
Flowchart of the study.

We evaluated the causal relationships between 731 immune cell phenotypes and HNMN using MR analysis. A flowchart of the study is shown in **Figure 1**. MR uses genetic variation to represent risk factors; therefore, meaningful IVs used in MR must meet three main presuppositions (1): IVs have a strong direct relationship with exposure (2), IVs do not correlate with possible confounders between exposure and outcome, and (3) IVs do not influence the outcome through pathways other than exposure. This study was conducted according to the Strengthening the Reporting of Observational Studies in Epidemiology Using Mendelian Randomization Guidelines (STROBE-MR, S1 Checklist) (15).

### 2.2 FinnGen data sources for HNMN

Genetic information on the HNMN was obtained from the FinnGen database. A total of 2,281 European cases and 314,193 European controls, amounting to 21,303,897 single-nucleotide polymorphisms (SNPs), were incorporated in the FinnGen database. The genetic data for HNMN are available at https://r10.finngen.fi/.

### 2.3 GWAS data sources for immune cells

We assessed the causal relationship between the 731 immune cell phenotypes and HNMN using MR. The data for each immune phenotype were available from the GWAS Catalog, with identification numbers ranging from GCST90001391 to GCST90002121 (16). The 731 immunophenotypes included absolute cell (AC) counts (n = 118), morphological parameters (MP) (n = 32), median fluorescence intensities (MFI) representing surface antigen levels (n = 389), and relative cell (RC) counts (n = 192). The MP trait comprised the conventional dendritic cell (CDC) and TBNK panels (T, B and NK cells). The MFI, RC and AC traits comprised TBNK, CDCs, myeloid cells, B cells, monocytes, Tregs, and mature stages of T cell panels (16).

The initial GWAS on immune traits employed data from 3,757 European individuals, and there were no overlapping cohorts (16). Following adjustment for covariates (such as sex and age), correlation studies were conducted on approximately 22 million SNPs using a Sardinian sequence-based reference panel (17).

### 2.4 Selection of IVs

Following recent studies (18, 19), SNPs associated with the immune trait significance threshold (P<1× 10-5) were selected as candidate IVs. SNP sites with low linkage disequilibrium (r2 < 0.01, aggregation window size = 500 kb) were selected using sample data from the European 1000 Genomes Project as a reference panel (20, 21). The proportion of phenotypic variation explained and the F-statistic were calculated for each IV to assess the IV strength and avoid weak instrumental bias. Calculation of the F-statistic consists of two steps: calculating R² and the F-value.


$$R^{2}=\frac{2\cdot \left(1-EAF\right)\cdot EAF\cdot \left(\beta^{2}\right)}{2\cdot \left(1-EAF\right)\cdot EAF\cdot \left(\beta^{2}\right)+2\cdot \left(1-EAF\right)\cdot EAF\cdot \left(SE^{2}\right)\cdot \mathrm{samplesize}}$$

In this context, EAF refers to the effect allele frequency, β represents the effect size of the exposure, SE is the standard error and sample size refers to the sample size. R² indicates the explanatory power of the model, reflecting the proportion of variance in the dependent variable (exposure) explained by the independent variable (SNP).


$$F=\frac{\left(\mathrm{samplesize}-2\right)\cdot R^{2}}{1-R^{2}}$$

IVs with F-statistics > 10 were regarded as strong IVs and reserved for further examination. Exposure and outcome SNPs were standardised to ensure consistent effect estimates for the same effect allele. Palindromic SNPs were also excluded (22). SNPs with a minor allele frequency (MAF) < 0.01 were also excluded (23). We searched the website (https://www.ebi.ac.uk/gwas/) and excluded SNPs associated with confounding factors such as PM2.5, smoking, alcohol consumption, and HPV infection (2, 19, 24).

### 2.5 Data analysis

We conducted MR analysis using the ‘TwoSampleMR’ package (version 0.5.8) in the R software environment (version 4.3.2). To explore the potential causal relationship between immune cells and HNMN, we used a variety of methods. Inverse-variance weighted (IVW), weighted median (WM), simple mode, MR-Egger, and weighted mode are among the numerous methods belonging to this category (25). The IVW method is an MR method used for meta-analysis of multiple locus effects when analysing multiple SNPs (26). This method provides more robust causal effect inference by weighting the effect estimates of multiple IVs, with weights equal to the inverse of their variances. This method is commonly employed for this purpose. WM is obtained by ranking all individual IV effect estimates according to their weights and then finding the median of the resulting distribution (27). When at least 50% of the information comes from valid IVs, WM can provide robust estimates. The simple mode method directly calculates the effects of each instrument variable and provides an overall causal effect estimate by aggregating these effects (27). In complex causal inference studies, it is necessary to combine other methods, such as the weighted mode or MR-Egger, to obtain more robust and reliable results. The weighted mode method calculates a weighted mode estimate (typically the median or weighted average) by weighting the effects of the instrument variables (28). It provides an estimate of the causal effect by identifying the patterns of all instrument variable effects. MR-Egger aims to assess causal relationships and test the validity of IVs. The MR-Egger method does not force the regression line to pass through the origin, allowing for an intercept term in the regression model (29). This enables it to account for IVs that exhibit directional pleiotropy. When the intercept is not zero and the p-value for the intercept is less than 0.05, it indicates the presence of pleiotropy. Heterogeneity assessments were conducted using MR-Egger and IVW methods (30). For results with heterogeneity, the multiplicative random effects inverse-variance weighting method (MREIVW) was used for MR analysis (31). The MR-Egger regression equation was utilised to evaluate the horizontal pleiotropy of IVs, whereby a p-value > 0.05 suggests the absence of notable pleiotropic effects (32). We applied an FDR correction owing to the increased likelihood of Type 1 errors with multiple testing (33). We conducted a leave-one-out sensitivity analysis of the key results to ascertain whether individual SNPs were responsible for the observed causal links. We used a scatter plot to show that the outliers had no effect on the results. We also used a funnel plot, which shows the robustness of the correlation in the absence of heterogeneity.

## 3 Results

### 3.1 Causal impact of immune cell phenotypes on HNMN

**Figure 2:**
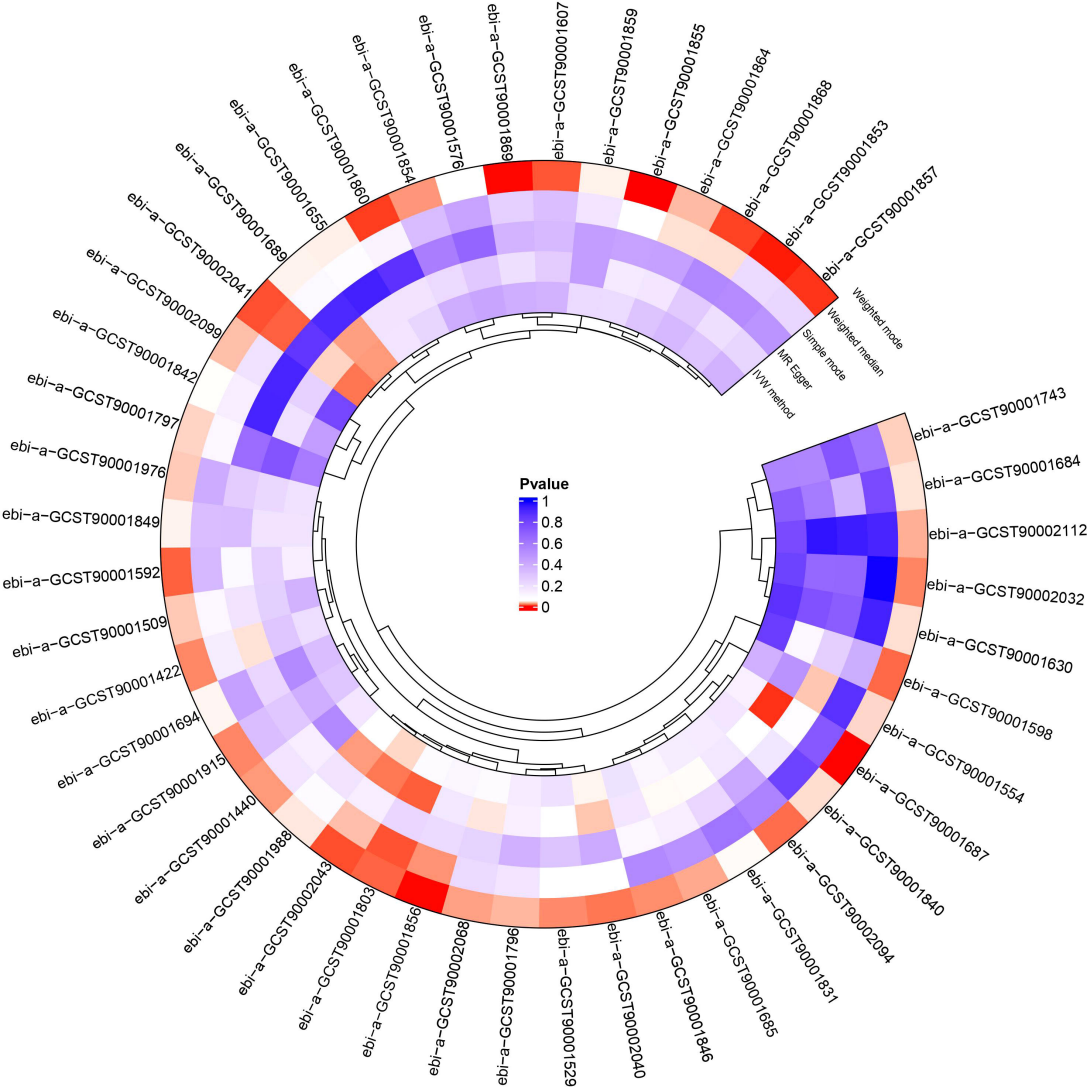
Circos heatmap of causal relationships between 731 immunophenotypes and the risk of HNMN.

We conducted a two-sample MR analysis to explore the causal relationships between the 731 immunophenotypes and HNMN (**Figure 2**; **Supplementary Tables S1**, **S2**). Four immune traits were identified with a significance of 0.1 after FDR was used. We explored the causal relationship between the 731 immune cell phenotypes and HNMN and conducted reverse MR. The results showed that HNMN had no causal relationship with the 731 immune cell phenotypes.

We detected significant associations of CD3 on secreting Tregs, CD3 on CD39+secreting Tregs, and CD3 on resting Tregs with a decreased risk of HNMN, while CD28− CD8 bright (br) AC retained a robust association with an increased risk of HNMN. The OR of CD28− CD8br AC (T cell panel) on the risk of HNMN was calculated to be 1.307 (95% confidence interval [CI] = 1.132–1.509, *P = 0.0003, P_fdr_ = 0.007*) using the MREIVW approach. CD3 on secreting Tregs exhibited a protective effect against HNMN (IVW: OR [95%]: 0.900 [0.854–0.950], *P = 0.0001, P_fdr_ = 0.077*). CD3 on CD39+secreting Tregs exhibited a protective effect against HNMN (IVW: OR [95%]: 0.903 [0.853 to 0.956], *P = 0.0004 P_fdr_ = 0.077*). CD3 on resting Tregs exhibited a protective effect against HNMN (IVW: OR [95%]: 0.924 [0.884–0.965], *P = 0.0004 P_fdr_ = 0.077*).

**Figure 3:**
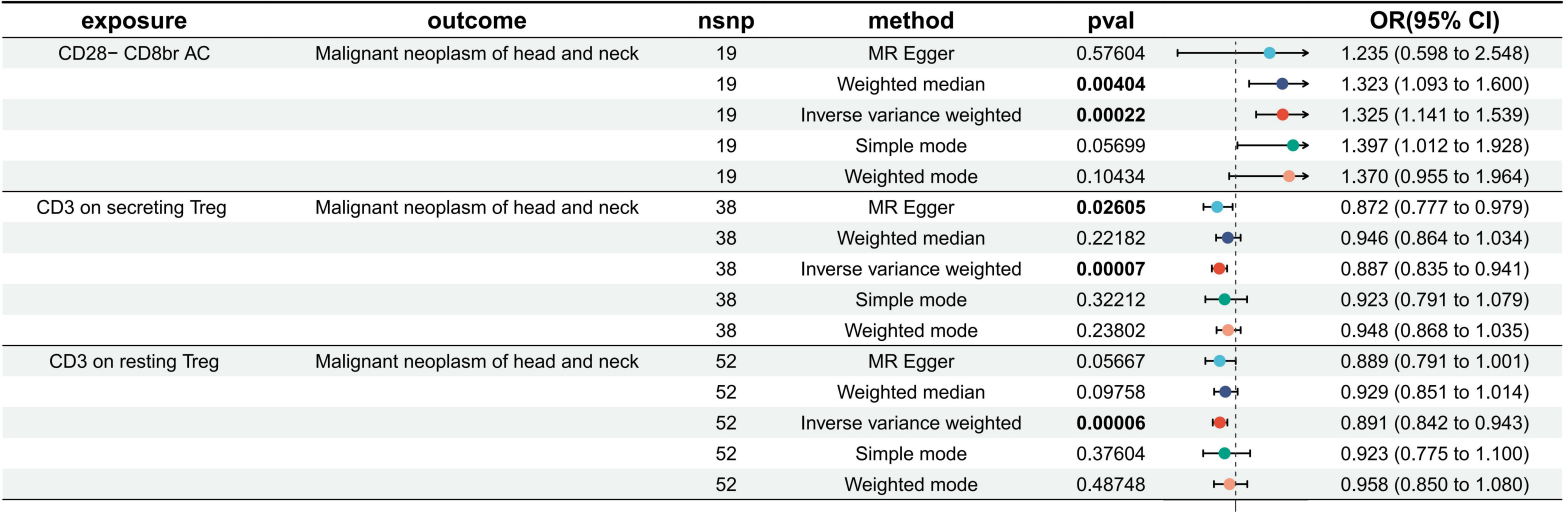
Effect of immune cells on HNMN. Nsnp, number of SNP; OR, odds ratio; CI, confidence interval.

**Figure 4:**
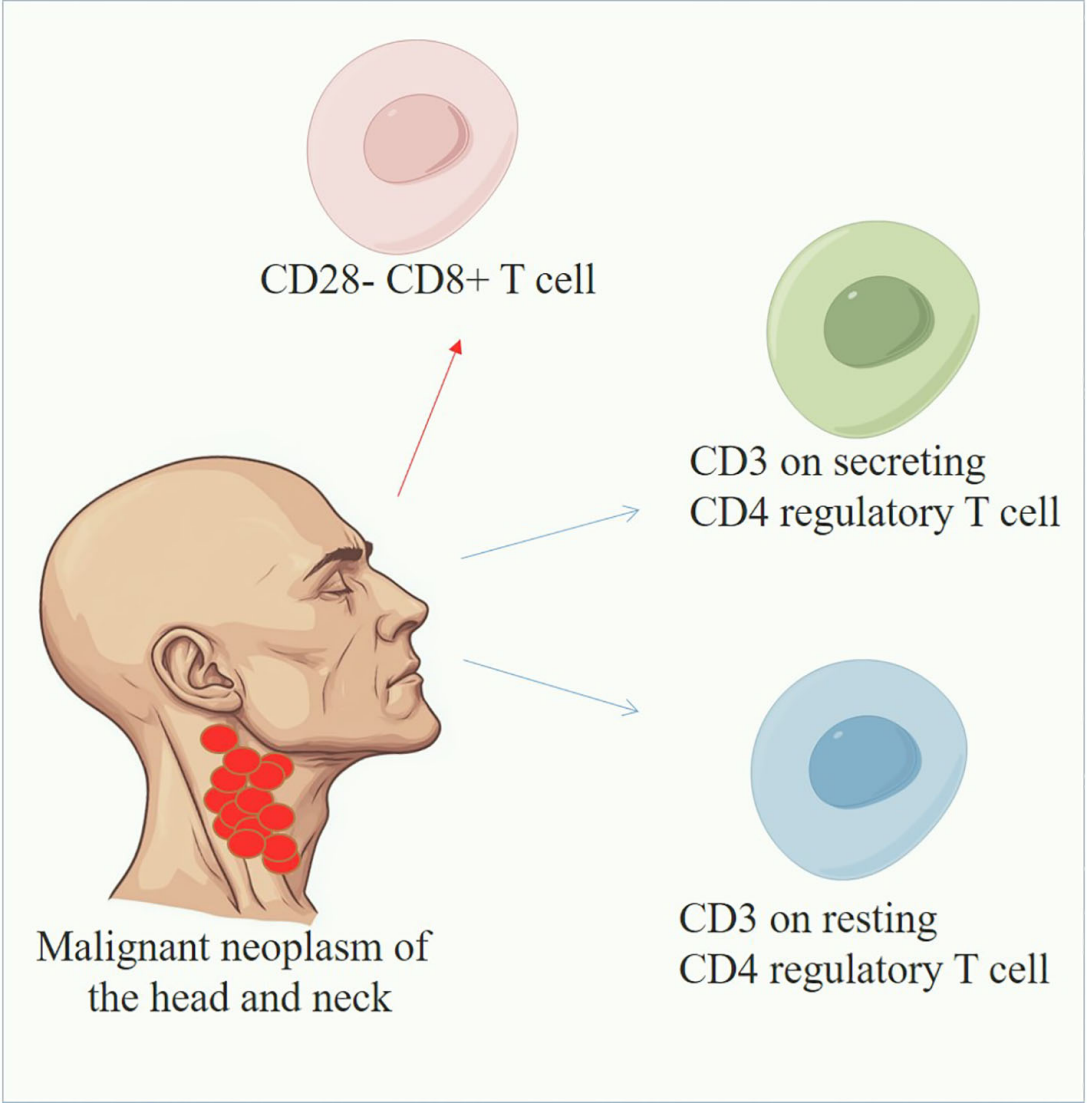
Overview of immune cells in HNMN. The blue line indicates that immune cells could reduce the risk of HNMN, whereas the red line indicates the opposite.

After excluding SNPs with an MAF < 0.01 and those associated with confounding factors, three immune phenotypes—CD3 on secreting Treg, CD3 on resting Treg, and CD28− CD8br AC—still showed robust associations with the risk of HNMN (**Figures 3**, **4**; **Supplementary Tables S3**, **S4**). The OR of CD28− CD8br AC (T cell panel) on the risk of HNMN was calculated to be 1.325 (95% CI = 1.413–1.539, P = 0.0002, Pfdr = 0.054) using the MREIVW approach. CD3 on secreting Tregs exhibited a protective effect against HNMN (IVW: OR [95%]: 0.887 [0.835–0.941], P = 0.00007, Pfdr = 0.025). CD3 on resting Tregs also exhibited a protective effect against HNMN (IVW: OR [95%]: 0.891 [0.842–0.943], P = 0.00006, Pfdr = 0.026). Additionally, although CD3 on CD39+ secreting Tregs exhibited a protective effect against HNMN (IVW: OR [95%]: 0.905 [0.851 to 0.962], P = 0.0013), the adjusted P value (Pfdr = 0.221) does not indicate significance.

### 3.2 Sensitive analysis

**Figure 5:**
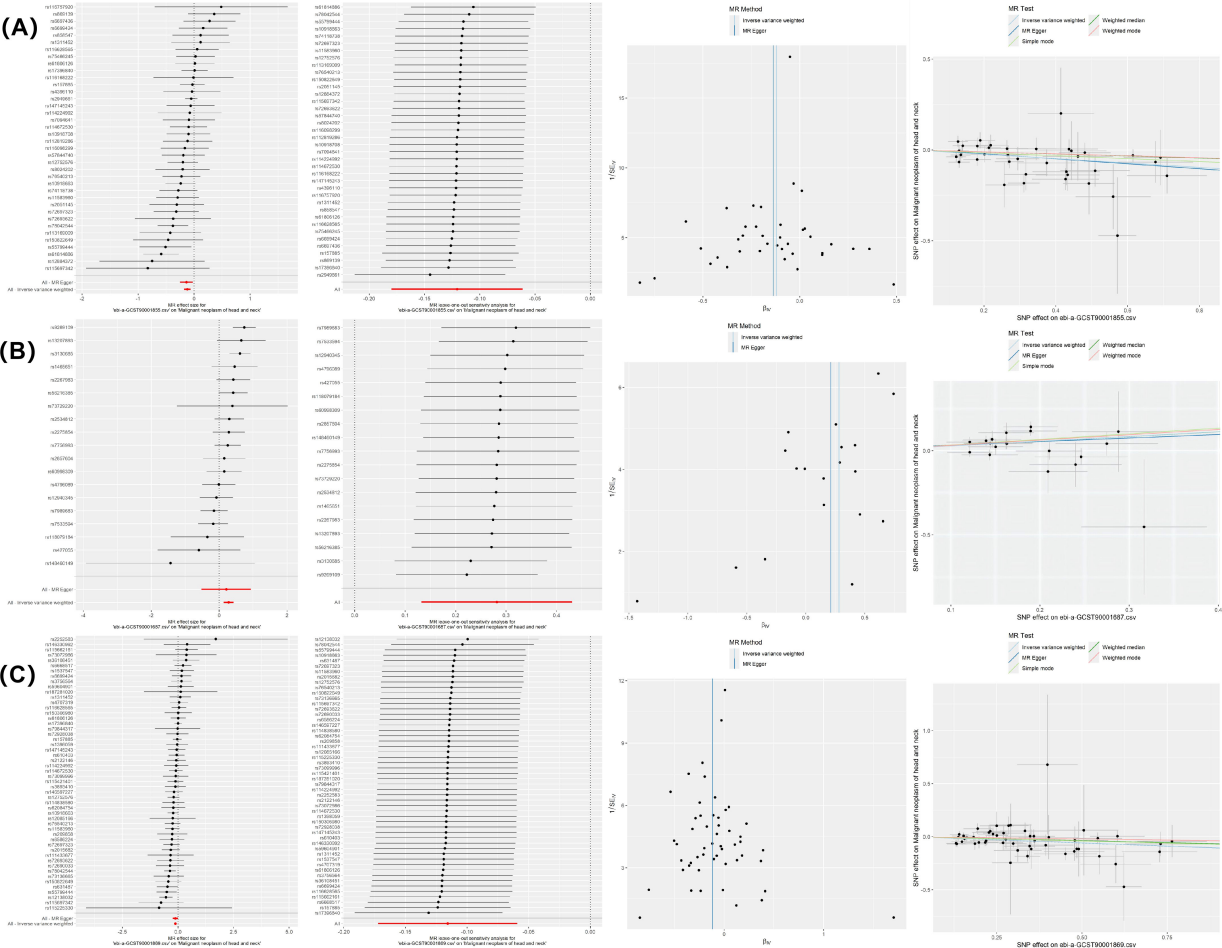
MR sensitivity analysis results. **(A)** CD3 on secreting Tregs on HNMN; **(B)** CD28− CD8br AC on HNMN; **(C)** CD3 on resting Tregs on HNMN.

The MR-Egger intercept and IVW test results indicated evidence of heterogeneity in the associations between CD28− CD8br AC and HNMN (*P < 0.05)*. Therefore, the MREIVW method was used for the MR analysis. There was no proof of heterogeneity in the correlation between the other two immune cell types and HNMN (*P > 0.05*). The Egger intercept test showed the absence of pleiotropy in the causal relationship between the three immune cell phenotypes and HNMN (*P > 0.05*). Leave-one-out, individual forest, scatter, and funnel plots confirmed the reliability of the results (**Figure 5**; **Supplementary Table S5**).

## 4 Discussion

Our research integrated large-scale individual and aggregated GWAS datasets to systematically examine the causal relationships between 731 immune cell traits and HNMN. Our study provides indicative findings that immune cells can affect the risk of HNMN using a comprehensive genetic method based on large-scale GWAS summary data. To the best of our knowledge, this is the first MR analysis that has explored the causal relationship between various immune cell phenotypes and HNMN. In this study, we verified that three immune cell types—CD3 on secreting Tregs, CD3 on resting Tregs, and CD28− CD8br AC—were significantly correlated with the risk of HNMN. The results showed that HNMN had no causal relationship with 731 immune cell phenotypes.

Our research revealed an association between elevated levels of CD3 on secreting Tregs and CD3 on resting Tregs and a decreased risk of HNMN. Tregs are a functionally mature subset of T cells that maintain peripheral immune tolerance and prevent excessive autoimmune reactions and chronic inflammatory diseases. Additionally, Tregs serve as suppressors of the antitumor response, promoting tumour immune escape through various mechanisms (34, 35). Increased infiltration of Tregs is associated with poor prognosis in a variety of cancer types such as non-small-cell lung cancer, renal cell carcinoma, and breast cancer (34). Nevertheless, research findings on the role of Tregs in the HNMN microenvironment and their relationship with tumour progression are inconsistent. A systematic review showed that the levels of FoxP3+ tumour-infiltrating lymphocytes in patients with HNSCC were positively correlated with patient survival prognosis. Foxp3 is the most specific and sensitive marker for identifying Tregs, implying that higher levels of Treg infiltration correlate with improved survival outcomes (36). Cho and Lim (37) revealed that high levels of circulating Tregs in the peripheral blood of patients with HNSCC can significantly increase disease-specific patient survival rates. Miyara et al. (38) divided FoxP3(+)CD4(+) T cells into three subsets based on their different phenotypes and functions: CD45RA(+)FoxP3(low) resting Tregs, CD45RA(−)FoxP3(high) activated Tregs, and cytokine-secreting CD45RA(−)FoxP3(low) non-suppressive T cells. Sun et al. (39) showed that the frequency of total Tregs in the peripheral blood of patients with HNSCC is higher than that in healthy individuals. Elevated levels of activated Tregs and cytokine-secreting Tregs were observed, whereas the frequency of resting Tregs decreased. Additionally, the frequency of activated Tregs and cytokine-secreting Tregs positively correlated with tumour progression. Activated Tregs significantly inhibit the proliferation of CD4+ CD25− T cells, suppress antitumor immunity, and promote immune escape (39). The secreted Treg subset can secrete large amounts of effector cytokines and does not exhibit suppressive activity *in vitro*, despite being significantly increased and associated with tumour progression in NHSCC. However, the function, differentiation and role of Tregs in HNSCC remain unclear and require further investigation. Our study revealed a correlation between elevated levels of CD3 on secreting Tregs, and CD3 on resting Tregs and a reduced risk of HNMN. Based on previous research, it is speculated that secreting and resting Tregs exhibit weaker immunosuppressive abilities than activated Tregs, potentially enhancing antitumor immunity. Owing to the limited coverage of the roles of various subtypes of Tregs in HNSCC, an in-depth exploration of the functions, differentiation and relationship with tumour progression of Treg subtypes, especially Tregs, in HNSCC and various subtypes of malignant tumours, will help provide new ideas for finding future immune-targeted therapy nodes.

Our research revealed an association between elevated levels of CD3 on Tregs and a reduced risk of HNMN. Rojo et al. (40) showed that Tregs expressing lower levels of TCR/CD3 chains (CD3ϵ,ζ), than CD4(+)CD25(−) Tconv. CD3ϵ chains in Tregs are highly concentrated in undegraded N-terminal sequences; in low pI isoforms, this trait is correlated with higher activation thresholds. Forced expression of mutant CD3ϵ chains lacking their N-terminal charges inhibits the differentiation of mature CD4+ T lymphocytes into Foxp3+ iTregs. Anti-CD3 antibodies that bind better to high pI CD3ϵ species increase the proportion of Tregs *in vivo*. CD3 on Tregs is associated with a higher activation threshold, potentially leading to enhanced antitumor immunity in the tumour microenvironment. This may explain the negative relationship between the expression of CD3 on Tregs and on HNMN. However, there are currently no reports of CD3 expression on Tregs in HNMN. Our findings provide new insights for further research on the role of CD3 on Tregs within the HNMN immune microenvironment.

T cell exhaustion and senescence result in functional impairment, leading to a reduced response to tumour antigens, which is a major mechanism of tumour immune escape (41). Our study revealed that CD28− CD8+ T cells were significantly associated with an increased risk of HNMN. CD28 is a crucial co-stimulatory molecule necessary for the activation of T cells that plays a critical role in the activation of CD8+ CTLs. Research has shown that the reduction of CD28 expression is a characteristic feature of senescent CD8+ T cells, and CD28− senescent T cells exhibit immunosuppressive functions in cancer (42). Di et al. (43) found that patients with microsatellite-stable cancer exhibited an immunosuppressive microenvironment in early tumour lesions, where CD8+ CD28− immunosenescent T cells with impaired proliferation capacity dominated the T cell population. Chen et al. (44) found that in patients with advanced non-small-cell lung cancer, the levels of circulating CD8+CD28− T cells are elevated and correlate with tumour burden and stage. The relationship between CD8+CD28− T cells and HNMN is similarly strong. Xu et al. (45) found that a high proportion of CD8+CD28− T cells in the circulating blood of patients with NPC before treatment was associated with a higher risk of disease progression and poorer survival rates. In their study, Fenoglio et al. observed decreased frequencies of CD8+CD28+ T cells and increased frequencies of both CD8+CD28− T lymphocytes and CD8+CD28−CD127−CD39+ Tregs in the group of patients with HNMN showing a poor response to therapy compared with those in patients showing a good response (46). This indicates that in HNMN, effector T cells are gradually exhausted and acquire regulatory properties, hindering their antitumor function. Research has shown that tumour-derived γδ Tregs can induce phenotypic changes in T cells, such as the downregulation of CD27 and CD28, leading to the senescence of responsive T cells (47). Blocking the Treg-induced senescence of effector T cells is crucial for reversing immune suppression in HNMN and will be a key focus of our future research.

SNPs are closely associated with the risk of HNMN. These variations may influence gene expression, thereby affecting key biological processes such as DNA damage, repair and drug absorption. Jelonek et al. (48) found differences in the SNPs of the DNA repair-related gene (XPD) between healthy controls and patients with HNMN. Laytragoon-Lewin et al. (49) found that SNPs in immune response genes and TNFα are associated with cancer risk and patient survival rates. Ahmed et al. (50) found that SNPs in the mitochondrial unfolded protein response pathway are associated with an increased risk of HNMN and may serve as predictive markers for the invasion and metastasis of HNMN. In addition, SNPs in HNMN are associated with sensitivity to chemoradiotherapy and radiotherapy-related complications. Farnebo et al. (51) found that SNPs in the DNA repair genes *XRCC3241* and *XPD751* affect the efficacy of cisplatin treatment in HNMN. Carles et al. (52) found that SNPs in multiple DNA repair genes significantly influence the response to radiation therapy in patients with HNMN. Similarly, Aguiar et al. (53) reported that several SNPs can be used to predict the risk of radiation dermatitis prior to the initiation of radiation therapy in patients with HNMN. Genes that may be influenced by SNPs and affect the risk and prognosis of HNMN include both non-coding and protein-coding genes, such as those identified in our research. Further in-depth research into the mechanisms of these SNPs will enhance our understanding of the pathophysiological processes of HNMN and provide a theoretical basis for precision medicine.

We performed MR analysis on data from a sizeable cohort comprising 316,474 individuals, which showed strong statistical power. Leveraging genetic IVs, we applied diverse MR analysis techniques to draw causal inferences, generating reliable results that addressed the issues of horizontal pleiotropy and confounding variables. This study has certain inherent limitations. First, the datasets do not encompass all ethnic groups. Given that the current GWAS datasets for immune cells are primarily derived from European populations, we selected European HNMN data from the FinnGen database to secure a consistent genetic background. This is the largest dataset available for HNMN. We hope that future research will include datasets from other populations to assess the generalizability of our findings to other ancestral groups. Second, the case group in our large cohort included only 2,281 European patients, which may result in insufficient statistical power for certain analyses and could, to some extent, affect the ability to draw causal inferences. Third, although we excluded rare variants and common confounding factors through SNP scanning and employed various statistical methods to control for potential confounders, our understanding of the relationship between SNPs and traits remains limited, which may introduce potential horizontal pleiotropy and heterogeneity, thereby weakening our ability to make causal inferences. Fourth, there is a complex biological interaction between HNMN and immune cells, and negative results may reflect this complexity rather than a true absence of association. Fifth, MR analysis only examined the causal relationship between exposure and outcome and could not clarify the specific mechanisms and potential mediating factors between immune phenotypes and HNMN. Sixth, we assumed a linear relationship between exposure and outcome, which may pose challenges in assessing potential non-linear relationships. Therefore, these results should be interpreted cautiously in the clinical context, and further research is needed to determine their clinical significance and the mechanisms underlying the interaction between immune cells and HNMN.

In conclusion, our MR analysis results indicate that CD3 on secreting Tregs and CD3 on resting Tregs are associated with a reduced risk of HNMN, whereas the presence of CD28− CD8+ T cells may be linked to an increased risk of HNMN. This indication provides new insights into the understanding of immune infiltration in the HNMN TME and opens up new opportunities for the development of immunotherapy drugs targeting checkpoint inhibitors of HNMN. In the future, it will be essential to expand the sample size to explore the causal relationships between immune cell phenotypes and various subtypes of HNMN to further validate and extend our findings. Additionally, integrating other omics data (such as transcriptomics and proteomics) will allow us to more thoroughly investigate how immune cell phenotypes influence the occurrence and progression of HNMN through specific mechanisms.

## Data Availability

Publicly available datasets were analysed in this study. This data can be found here: https://storage.googleapis.com/finngen-public-data-r10/summary_stats/finngen_R10_C3_HEAD_AND_NECK_EXALLC.gz and https://gwas.mrcieu.ac.uk/.
